# Phenotypic and genetic insights into efflux pump mechanism in *Mycoplasma anserisalpingitidis*

**DOI:** 10.3389/fmicb.2023.1216893

**Published:** 2023-07-11

**Authors:** Eszter Zsófia Nagy, Áron Botond Kovács, Enikő Wehmann, Katinka Bekő, Dorottya Földi, Krisztián Bányai, Zsuzsa Kreizinger, Miklós Gyuranecz

**Affiliations:** ^1^Veterinary Medical Research Institute, Budapest, Hungary; ^2^National Laboratory of Infectious Animal Diseases, Antimicrobial Resistance, Veterinary Public Health and Food Chain Safety, Budapest, Hungary; ^3^Department of Pharmacology and Toxicology, University of Veterinary Medicine, Budapest, Hungary; ^4^MolliScience Kft., Biatorbágy, Hungary; ^5^Department of Microbiology and Infectious Diseases, University of Veterinary Medicine, Budapest, Hungary

**Keywords:** *Mycoplasma* sp. 1220, efflux pump inhibitors, antimicrobial resistance, ABC efflux pump, minimal inhibitory concentration

## Abstract

**Introduction:**

*Mycoplasma anserisalpingitidis* is one of the most important waterfowl-pathogenic mycoplasmas. Due to inadequate antibiotic treatment, many strains with high minimal inhibitory concentration (MIC) values for multiple drugs have been isolated lately. Decreased antibiotic susceptibility in several *Mycoplasma* species are known to be associated with mutations in topoisomerase and ribosomal genes, but other strategies such as active efflux pump mechanisms were also described. The scope of this study was the phenotypic and genetic characterization of the active efflux mechanism in *M. anserisalpingitidis*

**Methods:**

We measured the MIC values in the presence and absence of different efflux pump inhibitors (EPIs), such as carbonyl cyanide m-chlorophenylhydrazine (CCCP), orthovanadate (OV), and reserpine (RSP). Moreover, bioinformatic tools were utilized to detect putative regulatory sequences of membrane transport proteins coding genes, while comparative genome analysis was performed to reveal potential markers of antibiotic resistance.

**Results:**

Out of the three examined EPIs, CCCP decreased the MICs at least two-fold below the original MICs (in 23 cases out of 36 strains). In the presence of OV or RSP, MIC value differences could be seen only if modified dilution series (10% decrease steps were used instead of two-fold dilutions) were applied (in 24/36 cases with OV and 9/36 with RSP). During comparative genome analysis, non-synonymous single nucleotide polymorphisms (nsSNPs) were identified in genes encoding ABC membrane transport proteins, which were displayed in higher percentages in *M. anserisalpingitidis* strains with increased MICs. In terms of other genes, a nsSNP was identified in DNA gyrase subunit A (*gyrA*) gene which can be related to decreased susceptibility to enrofloxacin. The present study is the first to highlight the importance of efflux pump mechanisms in *M. anserisalpingitidis*.

**Discussion:**

Considering the observed effects of the EPI CCCP against this bacterium, it can be assumed, that the use of EPIs would increase the efficiency of targeted antibiotic therapy in the future control of this pathogen. However, further research is required to obtain a more comprehensive understanding of efflux pump mechanism in this bacterium.

## 1. Introduction

*Mycoplasma anserisalpingitidis* is a bacterial pathogen infecting mainly goose, which can cause inflammation of the cloaca and genital tracts (Stipkovits et al., 1986; Hinz et al., 1994; Stipkovits and Kempf, 1996). In three-weeks-old waterfowl, peritonitis and airsacculitis can occur in association with the infection (Stipkovits et al., 1993). Mostly breeding flocks are exposed to this disease, where the morbidity can rise up to even 50–100%. In the affected flocks the egg production is significantly decreased and the embryo mortality can reach up to 40–60% (Dobos-Kovács et al., 2009). Consequently, this pathogen is responsible for significant economic losses (Stipkovits and Szathmary, 2012), which could only be prevented or treated by improving the housing conditions and using antibiotic therapy, as currently no vaccines are available commercially against *M. anserisalpingitidis*. However, many strains with high minimal inhibitory concentration (MIC) values for multiple drugs have been isolated from Europe and China lately (Grózner et al., 2016; Gyuranecz et al., 2020). So far three main mechanisms of antibiotic resistance have been described in bacteria: modification of target sites by methylation or mutation, drug inactivation and active efflux system (Giedraitienė et al., 2011). Bacterial efflux pumps are responsible for removing toxic substrates from the bacterial cell (Webber, 2003; Poole, 2007). Primary and secondary transporters have been described previously (Marquez, 2005). The group of primary transporters include the ATP-binding cassettes (ABC) and the ABC transporters, which use ATP as energy source. Members of the second group use the proton gradient as a source of energy and are classified into four further families: major facilitator superfamily (MFS), resistance nodulation and cell division family (RND), small multi-drug resistance family (SMR) and multi-drug and toxic compound extrusion family (MATE) (Marquez, 2005).

ATP-binding cassette (ABC) transporters represent one of the largest superfamilies of active membrane transport proteins. They all share the ability to bind and hydrolyze ATP to transport substrates across the lipid bilayer (Higgins, 1992). The primary functions of bacterial ABC transporters are nutrition uptake, as well as the elimination of drugs and metabolic waste (Schneider and Hunke, 1998; Dassa and Bouige, 2001). These transporters exhibit shared structural features, namely two hydrophobic transmembrane domains and two hydrophilic cytoplasmic domains (Linton and Higgins, 1998). Recently, a novel type of ABC transporter called Energy Coupling Factor (ECF) transporters, has been classified as significant in the uptake of micronutrients (Rodionov et al., 2009). The structure of the ECFs contains two nucleotide-binding domains (EcfA and EcfA’), a transmembrane domain (EcfT) and a substrate-binding component (EcfS).

Based on previous studies, efflux pumps have a vital role in low-level antibiotic resistance (Schmalstieg et al., 2012; Antunes et al., 2015). In case of *M. mycoides* subsp*. capri* significant differences were found between the MIC values in the presence or absence of orthovanadate (OV) in case of norfloxacin and ciprofloxacin, but only in strains originally inhibited by lower MICs (≤8 μg/mL for norfloxacin and ≤ 1 μg/mL for ciprofloxacin and enrofloxacin; Antunes et al., 2015). Similarly in *Mycobacterium* species, only low-level resistance could be inhibited by efflux pump inhibitors (Schmalstieg et al., 2012). Several reviews have recently been published which were dealing with the effect of different efflux pump inhibitors (Raherison et al., 2002; Antunes et al., 2015; Li et al., 2017). In these studies carbonyl cyanide m-chlorophenylhydrazine (CCCP), OV and reserpine (RSP) were used as primary efflux pump inhibitors. CCCP can interfere with the proton gradient, which results in the disruption of the ATP synthesis (Kasianowicz et al., 1984). OV inhibits the ATPase activity (Pezza, 2002), while RSP’s mechanism of action is the inhibition of ATP/Mg^2+^ pumps.

Multiple studies have been published that have identified mutations associated with antibiotic resistance in the coding regions (Lupien et al., 2013; Zwama and Nishino, 2021; Waldner et al., 2022) or the promoters (Kaatz et al., 1999; Unemo et al., 2019) of various efflux pump genes in other bacteria. In the mechanism of antibacterial resistance of mycoplasmas, despite the presence of other transport protein families, only the significance of ABC efflux pumps has been previously established (Pereyre et al., 2002; Raherison et al., 2005; Antunes et al., 2015; Chernova et al., 2016). Non-synonymous single nucleotide polymorphisms (nsSNPs) within the regions encoding ABC efflux genes have also been reported in cases of *Mycoplasma bovis* (Waldner et al., 2022) and *Mycoplasma pneumoniae* (Li et al., 2017). The current state of knowledge is limited regarding the regulation of the gene expression—including of ABC efflux genes—in mycoplasmas, because of the reduced genome and low percentage of genes involved in translation and transcription in these bacteria (Madeira and Gabriel, 2007). Previous studies have identified the positions of transcription factors, transcriptional start sites, and core promoter structure in Mollicutes (Lloréns-Rico et al., 2015; Fisunov et al., 2016; Yus et al., 2019), as well as several subsequences of ribosome binding sites (RBSs) in *Mycoplasma* species (Montero-Blay et al., 2019).

The scope of this study was to investigate the activity of ABC efflux pumps in *M. anserisalpingitidis* strains with increased MIC values for the most common antibiotics. Beside examining the effect of three different efflux pump inhibitors (CCCP, OV, RSP) on the MICs, comparative genome analysis was carried out to identify potential mutations involved in antimicrobial resistance. The examinations were focused on identifying non-synonymous mutations in genes previously reported to be associated with antibiotic resistance, as well as in coding genes and putative regulatory sequences of ABC transporter protein coding genes in *M. anserisalpingitidis*.

## 2. Materials and methods

### 2.1. Isolate selection

Eleven *M. anserisalpingitidis* clinical isolates were selected for the examinations based on differences in their phenotypic and genetic characteristics which has been previously described (Grózner et al., 2021; Table 1). In brief, the isolates were cultured in Oxoid Mycoplasma broth medium (Thermo Fisher Scientific, Inc./Oxoid, Inc., Waltham, MA) supplemented with Mycoplasma Supplement G (Thermo Fisher Scientific, Inc./Oxoid, Inc.), 0.5% (wt/vol) sodium pyruvate, 0.5% (wt/vol) glucose, 0.15% L-arginine hydrochloride and 0.05% (wt/vol) phenol red. MIC values of enrofloxacin (group of fluoroquinolones), lincomycin (from lincosamides), tiamulin (from pleuromutilins) and tilmicosin, tylosin, tylvalosin from the group of macrolides were determined with the broth micro-dilution method (Hannan, 2000). Based on the results of the MIC testing, three strains with high and three isolates with low MICs were chosen for each antimicrobial group for further analyses. Overall, six strains exhibited reduced antibiotic susceptibility to multiple widely used antibiotics: MycAv 47, 70 and 91 for enrofloxacin; MycAv 47, 67, and 68 for lincomycin and tiamulin; MycAv 67, 68, and 177 for macrolides. Additionally, five strains showed sensitivity to several antimicrobial agents: MycAv 63, 65, and 668 for enrofloxacin; MycAv 55, 63, and 668 for lincomycin; MycAv 50, 55, and 668 for tiamulin and macrolides (Table 1). The low and high MIC values were determined based on previous studies, as there are no official breakpoints available for veterinary mycoplasmas: low MIC value ≤0.5 μg/mL for enrofloxacin (Landman et al., 2008), tylvalosin (Behbahan et al., 2008; Ghaleh Gol et al., 2008); ≤2 μg/mL for lincomycin (Kempf et al., 1988); ≤1 μg/mL for tylosin (Landman et al., 2008); ≤8 μg/mL for tilmicosin (Landman et al., 2008); <0.625 μg/mL for tiamulin (Ghaleh Gol et al., 2008); high MIC value: ≥2 μg/mL for enrofloxacin (Landman et al., 2008); tylvalosin (Behbahan et al., 2008; Ghaleh Gol et al., 2008); ≥8 μg/mL for lincomycin (Kempf et al., 1988); ≥4 μg/mL for tylosin (Landman et al., 2008); ≥32 μg/mL for tilmicosin (Landman et al., 2008); ≥0.625 μg/mL for tiamulin (Ghaleh Gol et al., 2008). Whole genome sequences of the isolates were determined on Illumina platform (Illumina, Inc., San Diego, CA, United States) and multilocus sequence typing (MLST) was performed for the genetic characterization of the isolates previously (Grózner et al., 2021). The selected isolates belong to different clades based on MLST analysis (Table 1; Grózner et al., 2021).

**Table 1:** Background information about the isolates with high and low MIC values in this study.^a,b^

GenBank accession number	Strain ID	Initial MIC (μg/mL)						Clade	Subclade	Sample of origin
Type strain		Enrofloxacin	Lincomycin	Tiamulin	Tilmicosin	Tylosin	Tylvalosin			
CP042295	ATCC BAA-2147	0.312	1.000	0.312	≤0.250	≤0.250	≤0.250	C	3C	Phallus lymph
Field isolates with low MICs										
SRX9772795	MycAv 65	**1.250**	2.000	0.312	**≤**0.250	**≤**0.250	**≤**0.250	C	4C	Phallus lymph
SRX9772793	MycAv 63	**0.625**	**0.500**	0.156	64.000	4.000	**≤**0.250	C	6C	Tracheal swab
SRX7583695	MycAv 668	**0.312**	**≤0.250**	**0.156**	**≤0.250**	**≤0.250**	**≤0.250**	C	6C	Cloacal swab
SRX7583629	MycAv 55	2.500	**1.000**	**0.156**	**≤0.250**	**≤0.250**	**≤0.250**	C	4C	Ovarian follicles
SRX7583693	MycAv 50	10.000	4.000	**0.078**	**0.500**	**0.500**	**≤0.250**	B		Phallus
Field isolates with high MICs										
SRX7583639	MycAv 70	**5.000**	2.000	0.625	>64.000	4.000	**≤**0.250	B		Phallus lymph
SRX7583671	MycAv 47	**5.000**	**>128.000**	**0.625**	>64.000	4.000	1.000	C	6C	Lung, air sac
SRX7583648	MycAv 91	**5.000**	8.000	0.625	**≤**0.250	**≤**0.250	**≤**0.250	C	3C	Phallus
SRX7583635	MycAv 67	5.000	**>128.000**	**0.625**	**>128.000**	**>128.000**	**4.000**	C	1C	Phallus lymph
SRX7583636	MycAv 68	2.500	**>128.000**	**0.625**	**>128.000**	**>128.000**	**4.000**	C	1C	Phallus lymph
NZ_CP041664.1	MycAv 177	2.500	2.000	0.625	**>128.000**	**>64.000**	**4.000**	B		Phallus

Field isolates with low MICs are the parent strains of the *in vitro* cultivated mutants.

^a^Isolates with initial MIC values highlighted in bold were submitted to efflux pump inhibition tests with the given antibiotics.

^b^The classification to different clades and subclades are according to Grózner et al. (2021).

### 2.2. Preparation of *in vitro* mutants of low susceptibility to antibiotics

A total of 18 *in vitro* cultivated mutants were selected by serial passages from field isolates inhibited by lower MIC values for the examined antibiotics (Sulyok et al., 2017). The parent strains, the applied antibiotics and the 18 *in vitro* cultivated mutants can be seen in Supplementary Dataset S1. Briefly, two-fold dilution series were made from each antibiotic in sub-inhibitory concentrations for every selected parent strain with low MICs (listed in Table 1 and Supplementary Dataset S1). Enrofloxacin, lincomycin, tiamulin, tilmicosin, tylosin were produced by Vetranal^®^ Sigma-Aldrich, USA and tylvalosin was originated from ECO^®^ Animal Health Ltd., United Kingdom. Stock solutions were diluted in the concentration of 1 mg/mL and aliquots were stored at −70°C. Two-fold dilutions of each antibiotic were freshly prepared in Oxoid Mycoplasma broth medium (Thermo Fisher Scientific) in the range of 0.039–0.156 μg/mL for enrofloxacin and tiamulin, 0.125–0.5 μg/mL for lincomycin and macrolides in the first dilution panel. The selected isolates were incubated in Oxoid Mycoplasma broth medium (Thermo Fisher Scientific) at 37°C for 5 days, and the *M. anserisalpingitidis* cultures changing color (the red media turned yellow) at the highest antibiotic concentrations were inoculated into a fresh, two-fold dilution panel of the antibiotics, with increased concentrations. The serial passages were continued until the strains’ MICs reached above the sensitive MIC value determined by previous studies: ≥0.5 μg/mL for tylvalosin (Ghaleh Gol et al., 2008) and enrofloxacin (Landman et al., 2008), ≥1 μg/mL for tylosin (Landman et al., 2008), ≥8 μg/mL for tilmicosin (Landman et al., 2008), ≥2 μg/mL for lincomycin (Kempf et al., 1988), and ≥0.625 μg/mL for tiamulin (Ghaleh Gol et al., 2008). However, two (MycAv 63 and 65) of the three selected parent strains’ (MycAv 63, 65, 668) MIC values for enrofloxacin were already above 0.5 μg/mL, hence it was decided to set a higher threshold of 2 μg/mL for this antibiotic (Landman et al., 2008). In order to reach these MIC values, the sensitive *M. anserisalpingitidis* parent strains (MycAv 50, 55, 63, 65, 668) were submitted to four, increasing dilution series of enrofloxacin, lincomycin, tiamulin, tilmicosin, tylosin, and five, increasing dilution series of tylvalosin supplied liquid medium. *In vitro* developed mutants were passaged once in antibiotic-free medium and the MIC values were determined again (Supplementary Datasets S1,S2).

### 2.3. MIC testing

Antimicrobial susceptibility was measured by using broth micro-dilution method as described by Hannan (2000). For the MIC testing, twofold dilutions of each antibiotic were freshly prepared in the range of 0.039–10 μg/mL for enrofloxacin and tiamulin and 0.25–64/128 μg/mL for lincomycin, tilmicosin, tylosin and tylvalosin.

*M. anserisalpingitidis* type strain (ATCC BAA-2147) and five field isolates (the parent strains of the *in vitro* cultivated mutant strains, MycAv 50, 55, 63, 65, 668; Table 1) were used as control and the cultures were adjusted in Oxoid Mycoplasma broth medium (Thermo Fisher Scientific) to 10^5^ color changing unit (CCU)/mL. The experiment was carried out on 96-well plate and every experiment was performed in duplicates. The plates were incubated at 37°C and were checked every day for a week or until they did not show further color changes (red to yellow shift) for 2 days. Initial MICs were measured when the growth control changed color.

### 2.4. Determining the adequate concentrations of the efflux pump inhibitors

Broth micro-dilution assay (Hannan, 2000) was used in order to determine the sensitivity of *M. anserisalpingitidis* to CCCP, OV, and RSP. The efflux pump inhibitors were produced by Vetranal^®^ Sigma-Aldrich, United States. Two-fold dilutions of each efflux pump inhibitor were freshly prepared in the range of 0.191–97.92 μg/mL for CCCP, 0.07–35.84 mM for OV and 0.005–1.28 mg/mL for RSP. *M. anserisalpingitidis* cultures were adjusted in Oxoid Mycoplasma broth medium (Thermo Fisher Scientific) to 10^5^ CCU/mL. The experiment was carried out on 96-well plate and every experiment was performed in duplicates. The plates were incubated at 37°C and were checked every day until they did not show further color changes (red to yellow shift). The lowest efflux pump inhibitor concentration at which no color change was detected was considered the lethal dose of the efflux pump. The adequate concentrations of the efflux pump inhibitors to be used in the MIC testing were determined to be at least four-fold below of the lethal concentrations of each efflux pump inhibitors (Raherison et al., 2002; Li et al., 2017).

### 2.5. MIC testing with efflux pump inhibitors

A modified broth micro-dilution method was used to examine the effect of efflux pump inhibitors (Hannan, 2000; Antunes et al., 2015; Li et al., 2017). The MIC values against the field isolates (MycAv 47, 70, 91 for enrofloxacin; MycAv 47, 67, 68 for lincomycin and tiamulin, MycAv 67, 68, 177 for macrolides) and *in vitro* cultivated mutant strains with decreased antibiotic susceptibility (MycAv 63-M1, 65-M1, 668-M1 for enrofloxacin, MycAv 55-M2, 63-M2, 668-M2 for lincomycin, MycAv 50-M6, 55-M6, 668-M6 for tiamulin, MycAv 50-M3, 55-M3, 668-M3 for tilmicosin, MycAv 50-M5, 55-M5, MycAv 668-M5 for tylosin, MycAv 50-M4, 55-M4, 668-M4 for tylvalosin) were measured for each antibiotic both in the presence and in the absence of efflux pump inhibitors. A modified dilution series was applied where 10% decrease steps were used instead of two-fold dilutions in order to make a more sensitive system, except for the tests with CCCP (Supplementary Dataset S2). In this case, two-fold dilutions were applicable (applied antibiotic ranges were the same as the ones used for MIC testing of the control strains) due to the strong effect on MIC values which had been experienced during the pilot studies (data not shown). The efflux pump inhibitors were used in the following concentrations: 0.765 μg/mL CCCP, 0.56 mM OV and 0.02 mg/mL RSP. The efflux pump inhibitors were freshly prepared before every experiment and every test was carried out on 96-well plate. The *M. anserisalpingitidis* type strain (ATCC BAA-2147, GenBank accession number CP042295) was used as a quality control. *M. anserisalpingitidis* cultures were diluted in Oxoid Mycoplasma broth medium in the concentration of 10^5^ CCU/mL. The plates were incubated at 37°C and checked after 16, 20, 24, 40, 44, 48, 64, 68, and 72 h of incubation, then on a daily basis for 1 week, or until they did not show further color changes for 2 days. Each experiment was performed in duplicates. Initial MICs were measured when the growth control showed color change.

Two-sample paired (Wilcoxon) signed rank test was used to ascertain whether there is significant difference between MIC values in the presence and absence of each tested efflux pump inhibitor. The (Wilcoxon) signed rank test was performed by R 4.2.1. software (R Core Team, 2022).

### 2.6. Whole genome sequence determination and analyses of possible antibiotic resistance-associated markers

Genomic DNA of the 18 *in vitro* cultivated mutants (listed in Supplementary Dataset S1) were extracted from 2 mL of logarithmic-phase cultures with the help of a commercial kit (QIAamp DNA Mini Kit, Qiagen Inc., United States) according to the manufacturer’s instructions. The whole-genome sequencing was carried out by using NextSeq 500 Illumina equipment (Illumina, Inc., San Diego, CA, United States). The short reads were assembled using SPAdes software version 3.15.4 (Bankevich et al., 2012). The annotation of the genomes was done using Prokaryotic Genome Annotation Pipeline (PGAP) software version 6.3 (Li et al., 2021). The newly assembled genome sequences (Accessibility of the SRA reads: BioProject ID: PRJNA912395) and the field isolates with high MICs were compared to the parent strains of the *in vitro* developed *M. anserisalpingitidis* mutants, and to the type strain (ATCC BAA-2147) with progressive MAUVE algorithm (Darling et al., 2004). SNPs, deletions and insertions were searched by Geneious Prime 2019.2.1 software (Kearse et al., 2012). The putative SNPs were manually reviewed and sorted based on their capability to cause synonymous or non-synonymous SNP (nsSNP). The functions of those genes which contain nsSNPs were examined with the help of CARD database (Alcock et al., 2019), NCBI nucleotide database (Sayers et al., 2022) and current literature (Google Scholar, using the following key words: “antibiotic resistance” and the gene name or functional group, for example: “*gyrA*” “antibiotic resistance”). The common nsSNPs were checked in all *M. anserisalpingitidis* strains with publicly available whole genome sequences and MIC data (Grózner et al., 2022).

### 2.7. Identifying putative regulatory regions of efflux pump genes

Putative regulatory sequences were identified using the Promotech software version 1.0 (Chevez-Guardado and Peña-Castillo, 2021) and the genome of the *M. anserisalpingitidis* type strain. In order to minimize the number of false positive promoters, the score threshold was suggested to be above 0.6 (Weber et al., 2012; Siqueira et al., 2014; Lloréns-Rico et al., 2015). As the regulatory regions of mycoplasmas are not fully characterized, we established a more stringent threshold for our investigation. Sequences located upstream of an efflux pump coding gene with a score of 0.8 or higher were considered potential regulatory regions involved in efflux pump regulation. The putative regulatory sequences’ promoter regions and ribosome-binding sites were determined based on the literature (Lloréns-Rico et al., 2015; Fisunov et al., 2016; Montero-Blay et al., 2019; Yus et al., 2019) and MEME suite software (Bailey et al., 2015). The Promotech-detected potential efflux pump regulatory regions were used as the input for the MEME motif-finding software to identify the putative Pribnow boxes in the sequences.

## 3. Results

### 3.1. Efflux pump activity testing of *Mycoplasma anserisalpingitidis* isolates

**Figure 1:**
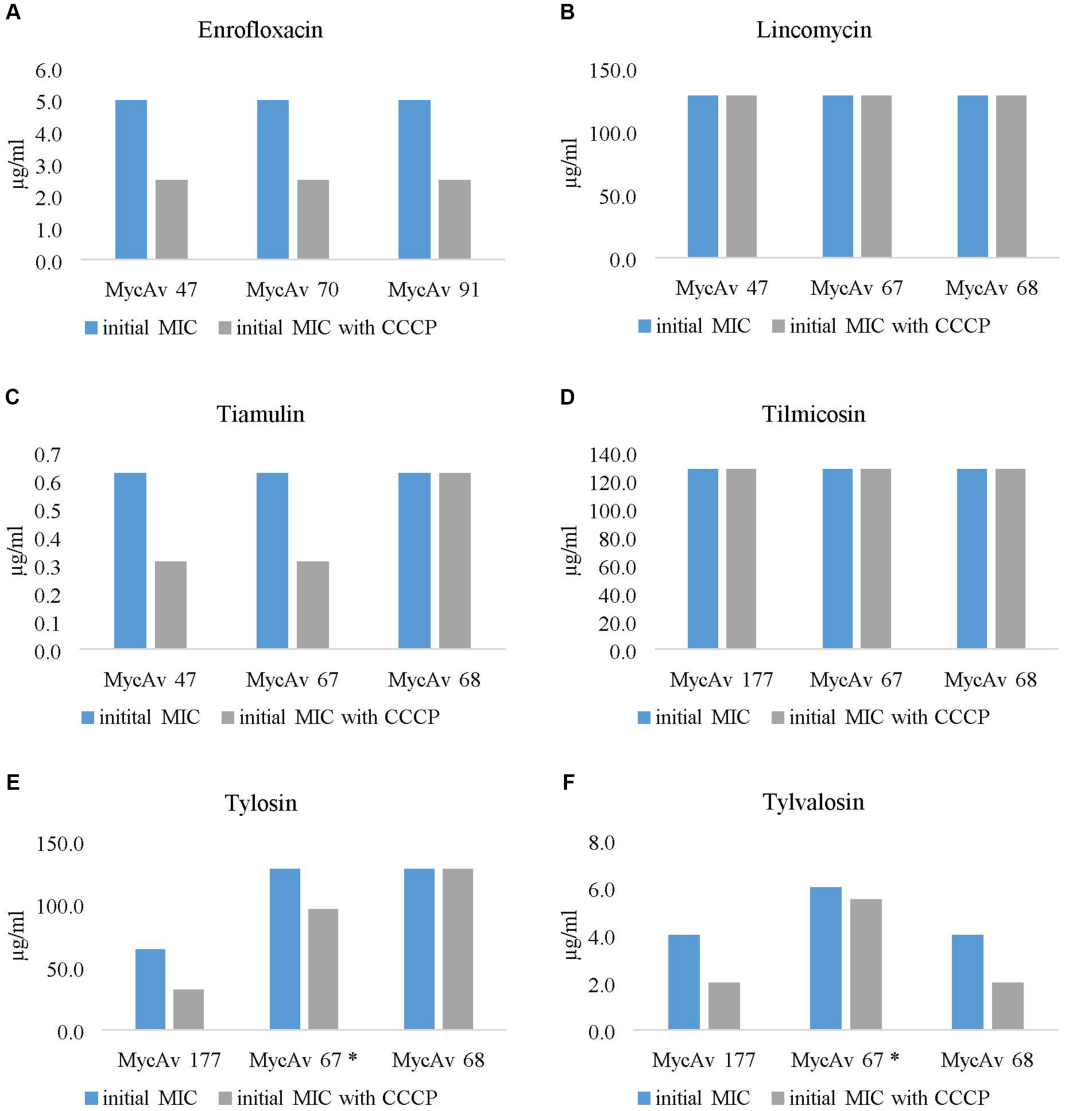
The effect of the most successful efflux pump inhibitor (carbonyl cyanide m-chlorophenylhydrazine (CCCP)) on the MICs of clinical isolates with decreased antibiotic susceptibility against enrofloxacin **(A)**, lincomycin **(B)**, tiamulin **(C)**, tilmicosin **(D)**, tylosin **(E)** and tylvalosin **(F)**. In the case of the MIC of MycAv 67 for tylosin and tylvalosin (marked with a star) could be seen differences only in modified dilution series (the concentration ranges used are presented in Supplementary Dataset S2).

**Figure 2:**
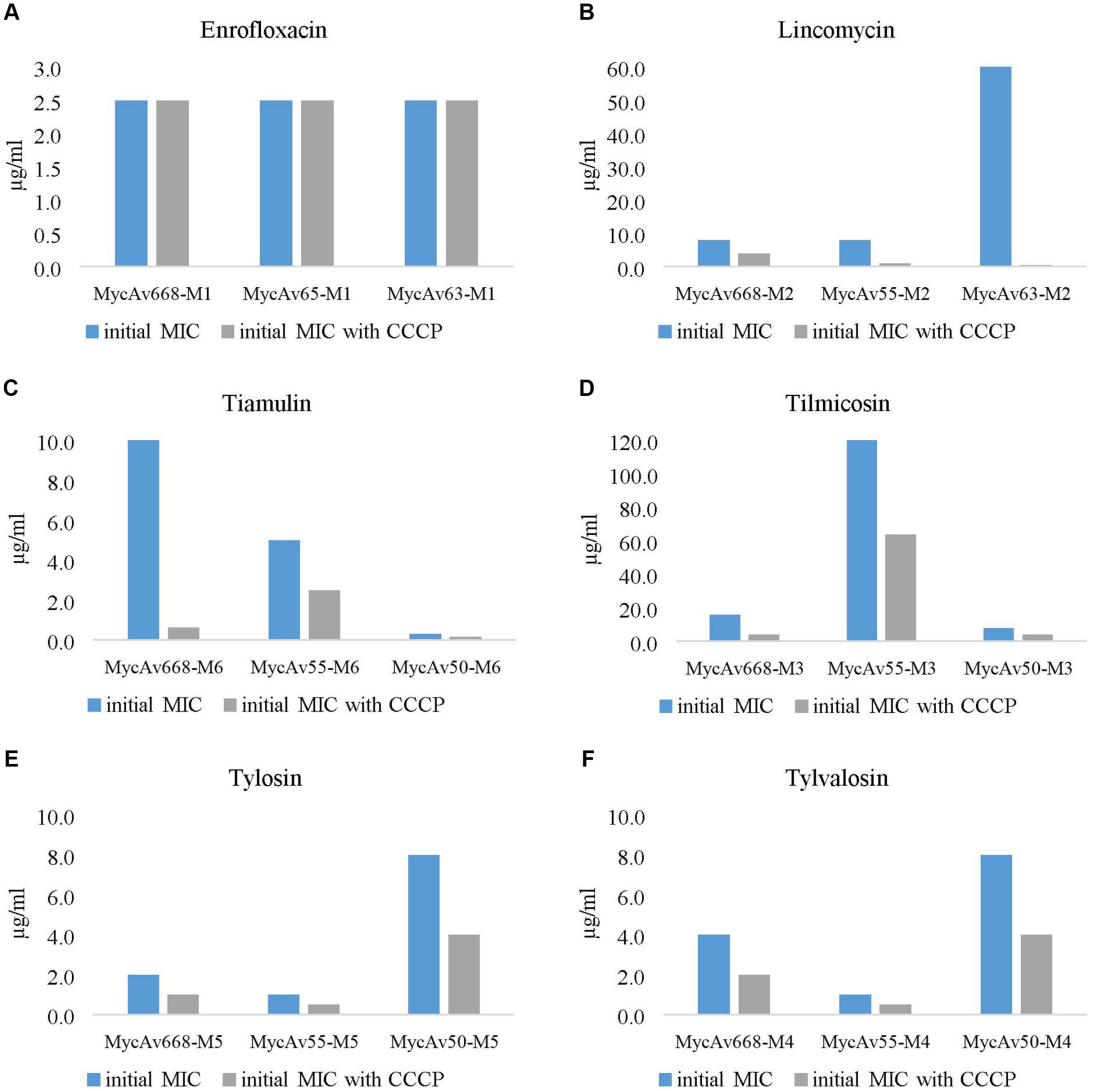
The effect of the most successful efflux pump inhibitor [carbonyl cyanide m-chlorophenylhydrazine (CCCP)] on the *in vitro* cultivated mutants’ MICs for enrofloxacin **(A)**, lincomycin **(B)**, tiamulin **(C)**, tilmicosin **(D)**, tylosin **(E)** and tylvalosin **(F)**. (the concentration ranges used are presented in Supplementary Dataset S2).

In this study initial MIC values of 18 *in vitro* cultivated mutants (MycAv 63-M1, 65-M1, 668-M1 for enrofloxacin, MycAv 55-M2, 63-M2, 668-M2 for lincomycin, MycAv 50-M6, 55-M6, 668-M6 for tiamulin, MycAv 50-M3, 55-M3, 668-M3 for tilmicosin, MycAv 50-M5, 55-M5, MycAv 668-M5 for tylosin, MycAv 50-M4, 55-M4, 668-M4 for tylvalosin) and six isolates with decreased antibiotic susceptibility (MycAv 47, 70, 91 for enrofloxacin; MycAv 47, 67, 68 for lincomycin, tiamulin, MycAv 67, 68, 177 for macrolides) were examined in the presence and in the absence of efflux pump inhibitors (Supplementary Dataset S2 and Figures 1, 2). In case of CCCP notable increases were detected in the efficiency of the antibiotics using twofold dilution series.

The MICs for enrofloxacin were halved in all cases out of the three field isolates tested (MycAv 47, 70, 91; Figure 1A); however, the *in vitro* cultivated mutants (MycAv 63-M1, 65-M1, 668-M1) did not show any detectable changes (Figure 2A). The MICs against the *in vitro* mutants with decreased lincomycin susceptibility (MycAv 55-M2, 63-M2, 668-M2) were reduced at least two-fold below (Figure 2B and Supplementary Dataset S2), while there could not be measured any changes in the MICs of clinical isolates of low susceptibility to this agent (MycAv 47, 67, 68 with >128 μg/mL MICs; Figure 1B). The MICs of *in vitro* mutants (MycAv 50-M6, 55-M6, 668-M6) and field isolates (MycAv 47, 67, 68) with decreased susceptibility to tiamulin dwindled at least two-fold below the original values, except in case of MycAv 68 clinical isolate with 0.625 μg/mL original MIC value (Figure 1C and Figure 2C). Regarding field isolates with increased MIC values for macrolides (MycAv 67, 68, 177), two-fold MIC decreases could be seen using CCCP in three cases: MIC value of MycAv 177 against tylosin and MycAv 177, 68 against tylvalosin. However, clinical isolates with extremely high MICs (MycAv 177, 67 with >128 μg/mL MIC for tilmicosin and MycAv 68 with >128 μg/mL MICs for tilmicosin and tylosin) did not show any changes (Figures 1D–F). Additionally, there was a field isolate (MycAv 67 in cases of tylosin and tylvalosin) where we could see differences only in modified dilution series (Supplementary Dataset S2). In cases of *in vitro* cultivated mutants with decreased susceptibility to macrolides, there were two- (MycAv 50-M3, MycAv 55-M3, MycAv 50-M4, MycAv 55-M4, MycAv 668-M4, MycAv 50-M5, MycAv 55-M5, MycAv 668-M5) or four-fold (MycAv 668-M3) reductions in the MIC values (Figures 2D–F and Supplementary Dataset S2). The difference was statistically significant between the MICs in the presence and absence of CCCP (in twofold dilution series) when all the mutants and field isolates were analyzed together (*p* < 0.001).

In the presence of OV or RSP MIC value differences could be seen only if modified dilution series were used (10% decrease steps were used instead of two-fold dilutions). There were detectable changes in 24 occasions out of the 36 examined combinations with OV (Supplementary Dataset S2), but these differences were not statistically significant. As for RSP, there were nine detectable, statistically not significant differences out of the 36 combinations (Supplementary Dataset S2).

### 3.2. Detection of possible antibiotic resistance markers

The 18 *in vitro* cultured mutants (listed in Supplementary Datasets S1, S2, BioProject ID: PRJNA912395) were compared to their parent strains (listed in Table 1 and Supplementary Dataset S1) to identify novel mutations associated with lower antibiotic susceptibility (Supplementary Datasets S3, S4). Furthermore, MycAv 47, 67, 68, 70, 91, and 177 (*M. anserisalpingitidis* strains with high MICs for several antibiotics, listed in Table 1) were compared to the *M. anserisalpingitidis* type strain (ATCC BAA-2147) and the sensitive parent strains (MycAv 50, 55, 63, 65, 668; listed in Table 1 and Supplementary Datasets S3, S4). ABC family genes that exhibited shared mutations in clinical isolates (or nsSNPs observed in both in clinical and *in vitro* mutants) were analyzed across all available *M. anserisalpingitidis* strains with publicly accessible whole genome sequences and MIC data (Supplementary Dataset S5).

Gene function predictions, which were based on *M. anserisalpingitidis* type strain (ATCC BAA-2147) gene annotation (GenBank accession number CP042295), showed that most SNPs were located in hypothetical proteins and intergenic regions. Numerous mutations were identified within genes that were previously described as antibiotic resistance-related genes. These genes encode ABC family members (Supplementary Dataset S3), 23S ribosomal RNA and a topoisomerase coding gene (Supplementary Dataset S4).

In the present study, numerous nsSNPs were identified in several members of the ABC family, which encompass diverse functions. Clinical isolates with high MIC values (MycAv 47, 67, 68, 70, 91, 177) exhibited nsSNPs within 14 genes that belong to the ABC family: WP_146368471.1, WP_146368786.1, WP_226364443.1, WP_146368361.1, WP_146368222.1, QDY87225.1, WP_146368766.1, WP_146368557.1, WP_146368360.1, WP_146368785.1, WP_146368574.1, WP_146368765.1, WP_146368337.1, WP_146368347.1 (Supplementary Dataset S3). The functional classification of the proteins was determined through *in silico* methods, utilizing Hidden Markov Model (HMM) or Subfamily Protein Architecture Labeling Engine (SPARCLE; Lu et al., 2020) employed by PGAP. Among these genes, WP_146368786.1, WP_146368785.1, and WP_146368557.1 are ABC transporter ATP-binding proteins that potentially serve as ATPase and permease components of an ABC-type multidrug transport system. WP_146368766.1 is an ABC transporter ATP-binding protein, likely functioning as the catalytic subunit of an ATP transporter complex involved in macrolide export. QDY87225.1 and WP_146368347.1 represent ATP-binding cassette domain-containing proteins with potential ATPase activity. Additionally, WP_146368361.1, WP_146368222.1, WP_146368360.1 are ABC transporter permease subunits, while WP_146368765.1 and WP_146368337.1 are ABC transporter permeases, indicating their probable involvement as permease components. Furthermore, nsSNPs were also found in two presumed ATP-binding genes (WP_146368471.1 and WP_226364443.1 ATP-binding cassette domain-containing proteins) and a potential energy-coupling factor transporter ATPase (EcfA, WP_146368574.1). Among these, five probable ABC transporter coding genes (NCBI reference: WP_146368786.1, WP_226364443.1, WP_146368361.1, WP_146368222.1, QDY87225.1) displayed one common mutation among isolates with decreased susceptibility to enrofloxacin, macrolides, lincomycin and tiamulin (Supplementary Datasets S3, S5).

Regarding *in vitro* cultivated mutants, nsSNPs were identified in three ABC protein coding genes: WP_146368461.1, which encodes an ABC transporter ATP-binding protein and is a potential ATPase component of an ABC-type multidrug transport system; WP_146367439.1, which encodes the energy-coupling factor transporter transmembrane protein EcfT (presumed T component of ECF-type transporters); and WP_146368471.1, an assumed ATP-binding gene encoding an ATP-binding cassette domain-containing protein. Specifically, MycAv 668-M1/2/3/4 exhibited nsSNPs within WP_146368461.1, and MycAv 65-M1 displayed nsSNPs within WP_146367439.1 (Supplementary Dataset S3). Furthermore, in the case of WP_146368471.1 ATP-binding cassette domain-containing protein nsSNPs were identified both in clinical isolates with decreased antibiotic susceptibility (MycAv 47, 67, 68, 70, 91, 177) and *in vitro* cultivated mutants (listed in Supplementary Dataset S3). In the field isolates (MycAv 47, 67, 68, 70, 91, 177) either one or both nsSNPs were found at the nucleotide positions 250 and 1,339 (according to the numbering of the concerned gene of the reference strain). On the other hand, in the *in vitro* cultivated mutants, strains with decreased susceptibility to lincosamide, tiamulin and macrolides (MycAv55-M2/3/4/5/6) exhibited a mutation at the nucleotide positions 786 and 789 in the same gene (Supplementary Dataset S3).

Those ABC transporter protein coding genes that displayed common mutations in clinical isolates (MycAv 47, 67, 68, 70, 91, 177) or both in clinical and *in vitro* developed mutants were examined in all *M. anserisalpingitidis* strains to see any possible patterns among field isolates with low and high MIC values (Table 2 and Supplementary Dataset S5). The examined nsSNPs could be detected in higher percentages in clinical isolates with decreased antibiotic susceptibility. Most prominent differences were observed in the cases of the ABC transporter ATP-binding protein (WP_146368786.1; a potential ATPase and permease component of an ABC-type multidrug transport system), the ABC transporter permease subunit (WP_146368222.1) and the ATP-binding cassette domain-containing protein (QDY87225.1) (Table 2 and Supplementary Dataset S5).

**Table 2:** The incidence rates of the non-synonymous single nucleotide polymorphisms (nsSNPs) in the ABC efflux pumps among strains with higher (High) and lower (Low) MIC values.^a^

Name of the protein	ATP-binding cassette domain-containing protein				ABC transporter ATP-binding protein		ATP-binding cassette domain-containing protein		ABC transporter permease subunit		ABC transporter permease subunit		ATP-binding cassette domain-containing protein	
Accessibility	WP_146368471.1				WP_146368786.1		WP_226364443.1		WP_146368361.1		WP_146368222.1		QDY87225.1	
nsSNP location	250		1,339		1,069		1,494		925		704		667	
	High	Low	High	Low	High	Low	High	Low	High	Low	High	Low	High	Low
Enrofloxacin	33.3% (8/24)	6.3% (1/16)	41.6% (10/24)	6.3% (1/16)	62.5% (15/24)	0.0% (0/16)	58.3% (14/24)	12.5% (2/16)	66.7% (16/24)	12.5% (2/16)	66.7% (16/24)	18.8% (3/16)	54.2% (13/24)	6.3% (1/16)
Tilmicosin	36.8% (7/19)	9.5% (2/21)	36.8% (7/19)	19.0% (4/21)	63.1% (12/19)	14.3% (3/21)	57.9% (11/19)	23.8% (5/21)	63.2% (12/19)	28.6% (6/21)	68.4% (13/19)	23.8% (5/21)	57.9% (11/19)	14.3% (3/21)
Tylosin	35.0% (7/20)	10.0% (2/20)	35.0% (7/20)	20.0% (4/20)	60.0% (12/20)	15.0% (3/20)	60.0% (12/20)	20.0% (4/20)	60.0% (12/20)	25.0% (5/20)	65.0% (13/20)	30.0% (6/20)	55.0% (11/20)	15.0% (3/20)
Tylvalosin	75.0% (3/4)	16.7% (6/36)	50.0% (2/4)	25.0% (9/36)	75.0% (3/4)	33.3% (12/36)	75.0% (3/4)	36.1% (13/36)	75.0% (3/4)	41.7% (15/36)	75.0% (3/4)	44.4% (16/36)	75.0% (3/4)	30.6% (11/36)
Lincomycin	60.0% (3/5)	17.1% (6/35)	80.0% (4/5)	20.0% (7/35)	100.0% (5/5)	28.6% (10/35)	80.0% (4/5)	34.3% (12/35)	100.0% (5/5)	37.1% (13/35)	100.0% (5/5)	40% (14/35)	80.0% (4/5)	28.6% (10/35)
Tiamulin	54.5% (6/11)	10.3% (3/29)	54.5% (6/11)	17.2% (5/29)	63.6% (7/11)	27.6% (8/29)	63.6% (7/11)	31.0% (9/29)	72.7% (8/11)	34.5% (10/29)	81.8% (9/11)	34.4% (10/29)	63.6% (7/11)	24.1% (7/29)

^a^Exact numbers of the strains that displayed the nsSNP out of every strain with higher and lower MIC values are shown in brackets.

Besides nsSNPs in ABC efflux pump coding genes, different mutations were identified in other genes previously associated with elevated MICs (Supplementary Dataset S4; Grózner et al., 2022). In terms of decreased susceptibility to fluoroquinolones, one common nsSNP was found in DNA gyrase subunit A (*gyrA*) gene [in 3/3 field isolates (MycAv 47, 70, 91) and in 2/3 (MycAv 63-M1, 668-M1) *in vitro* mutants] at the nucleotide position 446. Examining this mutation on all *M. anserisalpingitidis* strains with publicly available whole genome sequences and MIC data, there were three types of nsSNPs: strains with extremely high MICs (initial MICs were > 10 μg/mL) and eight strains with elevated MICs (six strains with initial MICs of 5 μg/mL, two strains of 2.5 μg/mL initial MIC values) had an nsSNP at the nucleotide position 446 which altered the original amino acid from Threonine to Isoleucine. Fourteen strains with increased MIC (initial MICs were 5 μg/mL in four cases and 10 strains had 2.5 μg/mL initial MIC value) and three strains with lower MIC (initial MICs were 1.25 μg/mL) displayed Guanine at the nucleotide position 445 which caused a changed from Threonine to Alanine. Strains in the third group were inhibited at lower MIC values (initial MICs were < 1.25 μg/mL) and contained Adenine and Cytosine at the nucleotide positions 445 and 446, coding Threonine (Supplementary Dataset S5).

Evaluating decreased tiamulin susceptibility, *in vitro* cultivated mutants (MyAv 50-M6, 55-M6, 668-M6) displayed mutations in the 23S rRNA gene at different positions (179, 434, 1,502, 1,568, 2,457) according to the numbering of the concerned gene of the type strain, while field isolates (MycAv 47, 67, 68, 70, 177) showed mutations at the nucleotide positions 793, 1,502 and 2067. In terms of the *in vitro* cultivated mutants with higher MIC values for lincomycin (MycAv 55-M2, 63-M2, 668-M2), mutations were determined in the 23S rRNA gene at different positions (179, 434, 1,568, 2067, 2,621). Regarding field isolates with decreased macrolide susceptibility (MycAv 67, 68, 177), three different nsSNPs were identified in the 23S rRNA gene at the nucleotide positions 793, 1,502 and 2067. The same nsSNP at the nucleotide position 793 was also present in three *in vitro* mutants (MycAv668-M3/5, MycAv55-M4), beside others (nucleotide positions 434, 544, 1,502, 1,568). For all strains of this bacterium with publicly available whole genome sequences and MIC data, the nsSNPs in the 23S rRNA gene were analyzed and reported in a previous work (Grózner et al., 2022).

### 3.3. Identifying putative regulatory regions of efflux pump genes

**Figure 3:**
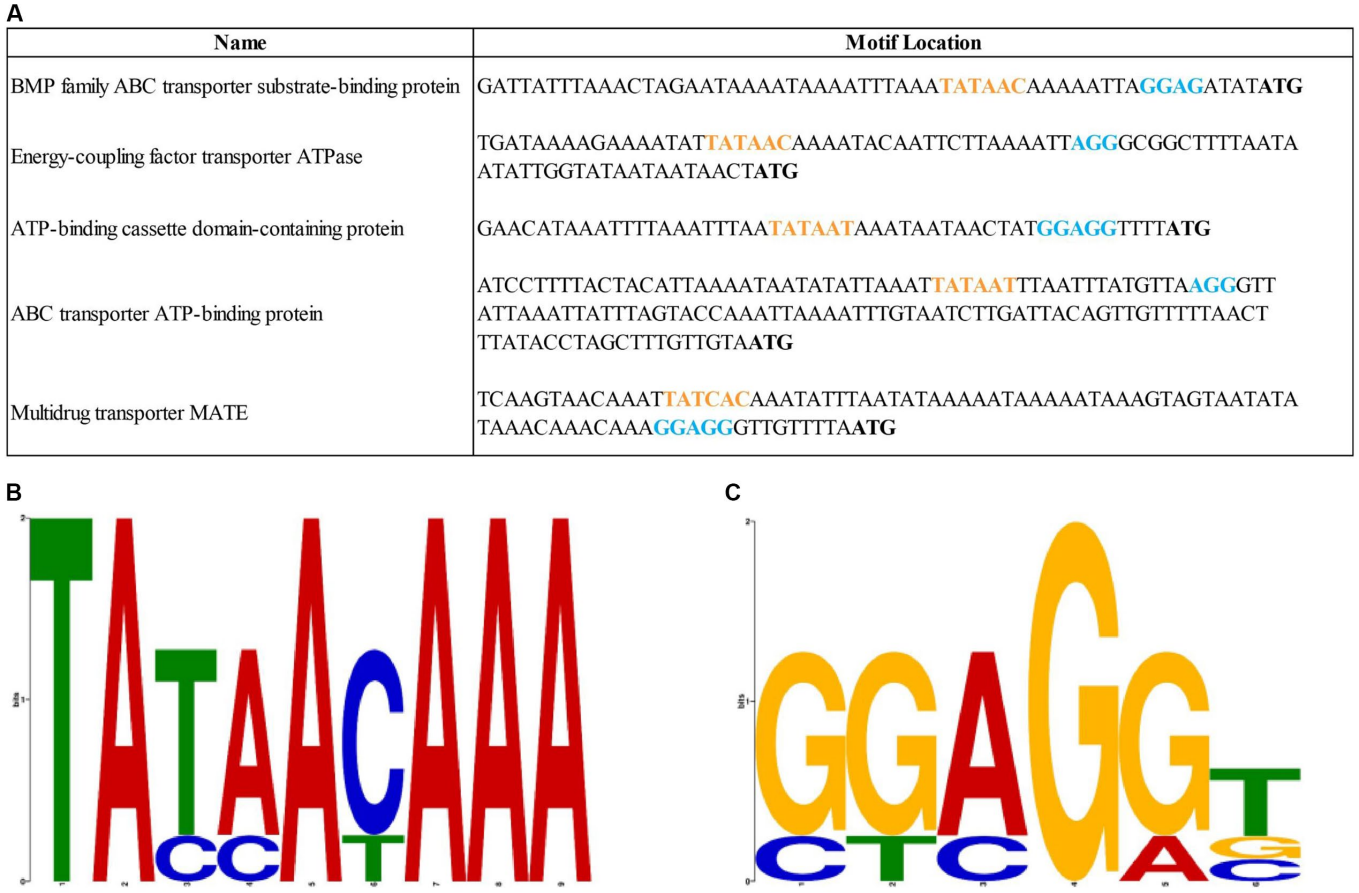
**(A)** The sequences of the putative regulatory regions of the efflux pumps in *M. anserisalpingitidis*. Orange letters show the potential Pribnow box, while blue letters represent the assumed ribosome binding sites. Start codon is written in bold. **(B)** The putative sequence of the Pribnow box based on MEME suite analysis. **(C)** The putative sequence of the ribosome binding sites based on MEME suite analysis. Each stock of letter in the MEME suite logos **(B,C)** represents a position in the motif. The bit score shows the similarity of the sequences, where a higher bit score indicates a higher similarity. The height of the individual letters within a stack is determined by the possibility of the letter at that position.

Using the Promotech software, five putative efflux pump regulatory regions were identified related to the following genes: BMP family ABC transporter substrate-binding protein (NCBI reference: QDY86627.1), energy-coupling factor transporter ATPase (QDY86993.1), ATP-binding cassette domain-containing protein (WP_226364443.1), ABC transporter ATP-binding protein (WP_146368785.1), and multidrug transporter MATE (WP_201798414.1). The identified putative regulatory sequences with potential Pribnow box and RBSs (Figure 3A) showed statistically insignificant *E-*values according to the MEME analyses (*E*-value of the potential RBSs sequences is 0.33 and the *E*-value of the assumed Pribnow box is 2.4; Figures 3B,C). The putative efflux pump regulatory regions of the *M. anserisalpingitidis* type strain were compared to those of all *M. anserisalpingitidis* strains with publicly available whole genome sequences. However, no significant nsSNPs were found in these regions that could potentially contribute to lower antibiotic sensitivity.

## 4. Discussion

*M. anserisalpingitidis* is a common pathogen of geese and it can cause significant economic losses (Dobos-Kovács et al., 2009). Several strains with decreased antibiotic susceptibility were isolated in the past decade (Grózner et al., 2016; Gyuranecz et al., 2020), which is most probably the consequence of abundant antibiotic usage in the control of *M. anserisalpingitidis* and/or other bacterial infections. The observed high rate of decreased antibiotic susceptibility might be connected with the high genetic variability of this species and capability to rapidly develop resistance to antimicrobials (as was seen in the present study during the development of the mutants with decreased antibiotic susceptibility). In microbes with slightly increased MIC values, the efflux pump mechanism can play an essential role (Schmalstieg et al., 2012), expelling the antibiotics before they reach their targets. Active efflux pump mechanism was identified both in Gram positive and Gram negative bacteria (Van Bambeke et al., 2000), including different *Mycoplasma* species.

The effects of efflux pump inhibitors were also reported in several *Mycoplasma* species formerly (Pereyre et al., 2002; Antunes et al., 2015; Li et al., 2017). In our research, the determination of the MIC values in the presence and absence of different EPIs revealed statistically significant differences when CCCP was applied. The differences between the effectiveness of the particular EPIs can be explained by their diverse working mechanism. The efflux pump inhibitors can be effective on different types of (primarily and secondarily) efflux pumps, especially the CCCP which can interfere with the proton gradient. This results in the disruption of the ATP synthesis (Kasianowicz et al., 1984) and the inhibition of the proton motive force (PMF) (Pagès et al., 2005). Based on these findings, further examination of the secondary efflux pumps in *M. anserisalpingitidis* can be an area for future research.

In the case of certain strains with extremely high MICs, none of the efflux pump inhibitors were effective (there were seven cases where none of the EPIs were effective out of 11 occasions where *M. anserisalpingitidis* strains have extremely high MICs). In these cases, the lack of detectable effect of the EPIs is explained by that efflux pump mechanism is the first step in the development of antibiotic resistance (Antunes et al., 2015). According to Schmalstieg and co-workers the increasing activity of efflux pumps is a direct response to the sub-inhibitory concentrations of different antibiotics (Schmalstieg et al., 2012). Under the protection of efflux pumps, bacteria can continue replicating and the possibilities of genome mutations are increasing. Eventually, higher-level resistance can emerge due to the mutations. Based on this model, the increasing activity of efflux pumps and the development of higher-level resistance are ordered molecular events in the process of acquiring antibiotic resistance. Additionally, in isolates with high MIC values in the present study (Supplementary Dataset S4) nsSNPs could be identified which were previously described as antibiotic resistance-related markers in *M. anserisalpingitidis* [in the 23S rRNA gene at the nucleotide positions 793 and 2067 (Grózner et al., 2022)], or which were in genes what contain previously described antibiotic resistance-related markers [DNA gyrase subunit A (*gyrA*) at the nucleotide position 446; Sulyok et al., 2017]. When compared to the *M. anserisalpingitidis* type strain, the majority of *M. anserisalpingitidis* strains displayed significantly elevated MIC values for enrofloxacin and macrolides, with notable increases observed for tilmicosin and tylosin. This can be explained by the detected mutations in the target genes of these antibiotics (*gyrA* for enrofloxacin and the 23S rRNA for macrolides).

The role of the genes of ABC efflux pumps in the mechanism of antibacterial resistance in mycoplasmas has been previously described (Pereyre et al., 2002; Raherison et al., 2002, 2005; Antunes et al., 2015; Chernova et al., 2016), in contrast to other families of transport proteins. Furthermore, nsSNPs have been reported in the ABC efflux gene encoding regions of *M. bovis* (Waldner et al., 2022), *M. pneumoniae* (Li et al., 2017), and *Streptococcus pneumoniae* (Lupien et al., 2013), as well as in the RND pumps of other bacterial species such as *Acinetobacter baumannii, Escherichia coli, Legionella pneumophila, Neisseria gonorrhoeae, Pseudomonas aeruginosa*, and *Salmonella enterica* (Zwama and Nishino, 2021). SNPs related to antibiotic resistance have also been described in the promoter regions of efflux pumps, specifically in *N. gonorrhoeae* and *Staphylococcus aureus* (Kaatz et al., 1999; Unemo et al., 2019).

Therefore, the present study focused on conducting further comparative genome analysis specifically genes belonging to the ABC superfamily. Based on NCBI database, the *M. anserisalpingitidis* type strain was found to possess a minimum of 37 genes identified as members of the ABC family. Extensive investigation in this study unveiled numerous nsSNPs within various members of the ABC family, each with distinct functional roles. In total, 14 putative ABC genes were found to harbor nsSNPs in clinical isolates exhibiting reduced antibiotic susceptibility (MycAv 47, 67, 68, 70, 91, and 177), while three potential ABC family members were identified in the context of *in vitro* cultivated mutants. Specifically, clinical isolates sensitivity (MycAv 47, 67, 68, 70, 91, 177) exhibited nsSNPS in WP_146368786.1, WP_146368785.1, WP_146368557.1, which are postulated to be probable ATPase and permease components of an ABC-type multidrug transport system. Furthermore, a potential ATPase catalytic subunit of an ATP transporter complex involved in macrolide export (WP_146368766.1) displayed nsSNPS in the aforementioned clinical isolates. In the case of *in vitro* cultured mutants, nsSNPs were discovered in a presumed component of an ABC-type multidrug transport system (WP_146368461.1).

Additionally, our investigation identified nsSNPs in the energy-coupling factor transporter ATPase (EcfA, WP_146368574.1) and the energy-coupling factor transporter transmembrane protein EcfT (T component of ECF-type transporters, WP_146367439.1). While ECF transporters are primarily recognized for their involvement in micronutrient transport, the potential significance of energy-coupling factor transporter transmembrane protein EcfT in antibiotic resistance has been reported in *M. bovis* (Ledger et al., 2020) and tigecycline resistant *Enterococcus faecalis* (Bai et al., 2022).

Furthermore, certain ABC protein coding gene pairs (WP_146368360.1 and WP_146368361.1, WP_146368765.1 and WP_146368766.1, and WP_146368785.1 and WP_146368786.1) were found to overlap. It is well known that mycoplasmas possess small genomes (less than 1 Mbp), and overlapping genes are believed to be a consequence of evolutionary pressure to reduce the genome size. Previous studies have reported 162 overlapping gene pairs in *M. genitalium* and 203 in *M. pneumoniae* genomes (Fukuda et al., 1999). Additionally, two overlapping ABC protein coding genes associated with ciprofloxacin resistance have been documented (Raherison et al., 2005). Functional roles have been observed for several overlapping genes in prokaryotes (Chen et al., 1990; Inokuchi et al., 2000; Fukuda et al., 2003). However, the gene structure in this context was a result of incidental elongation of coding regions in *M. genitalium* and *M. pneumoniae* (Fukuda et al., 1999). Further research is necessary to fully comprehend the significance of these overlapping ABC protein coding gene in *M. anserisalpingitidis*.

Upon examining the identified nsSNPs in all *M. anserisalpingitidis* strains with publically available whole genome sequences, we found that the nsSNPs were present in higher percentages in field isolates with high MICs, although an exact correlation with the high MIC values could not be established. During this study, we did not examine the consequences of these mutations, such as the possible overexpression of the gene or increased function of the efflux pump, and none of them have been identified so far as potential single-drug resistance (SDR) or multidrug resistance (MDR) marker in mycoplasmas. To ascertain the significance of their role in antibiotic resistance, additional investigations are necessary, including the analysis of gene regulation. Nevertheless, considering that SNPs can occur also in the regulatory elements, we identified putative regulation sequences of the efflux pumps using bioinformatics tools. However, during the comparative genome analysis, no SNPs associated with antibiotic resistance were detected in these regions and E-values of the described Pribnow box and RBSs were not significant either. It is worth mentioning though, that MEME E-values may underestimate the true significance when the input dataset consists of few sequences (specifically, only five in this case; Bailey et al., 2015).

This study revealed active efflux mechanism in *M. anserisalpingitidis* strains for the first time. Efflux pump inhibitors are naturally-generated or synthetic molecules that can inhibit efflux pumps. Utilizing them as adjuvants can restore the effectiveness or increase the potency of antimicrobial agents (Pagès and Amaral, 2009). Although EPIs are great opportunities to fight against antibiotic resistance, the development of commercial EPIs is challenging (AlMatar et al., 2021), and their clinical use warrants further studies. Beside their capability to inhibit the expulsion of most of the clinically important antibiotics from the intracellular environment, there are recent publications about their role in the inhibition of biofilm formation by interfering in the process of releasing the quorum sensing molecules by efflux pumps which facilitate biofilm matrix formation (AlMatar et al., 2021). Based on previous studies (Bekő et al., 2022), biofilm formation can assist *M. anserisalpingitidis* to survive in the environment. Considering the observed effects of the EPI CCCP against *M. anserisalpingitidis* in the present study, and the probable interference with biofilm formation, it is assumed, that the use of EPIs would increase the efficiency of targeted antibiotic therapy in the control of this pathogen.

## Data availability statement

The datasets presented in this study can be found in online repositories. The names of the repository/repositories and accession number(s) can be at: https://www.ncbi.nlm.nih.gov/genbank/, BioProject ID: PRJNA912395.

## Author contributions

EN, EW, KBe, DF, ZK, and MG contributed to conception and design of the study. ÁK, EW, and EN performed the genomic and statistical analysis. KBá carried out the whole genome sequencing. EN wrote the first draft of the manuscript. All authors contributed to manuscript revision, read, and approved the submitted version.

## Funding

This work was supported by the KKP19 (129751) grant of the National Research, Development and Innovation Office, Hungary, the SA-27/2021 grant of the Eötvös Loránd Research Network, the Project no. RRF-2.3.1-21-2022-00001 which has been implemented with the support provided by the Recovery and Resilience Facility (RRF), financed under the National Recovery Fund budget estimate, RRF-2.3.1–21 funding scheme and the support provided by the Ministry of Innovation and Technology of Hungary (legal successor: Ministry of Culture and Innovation of Hungary) from the National Research, Development and Innovation Fund, financed under the TKP2021-EGA-01 funding scheme of the National Research, Development and Innovation Office. The funders had no role in study design, data collection and interpretation, or the decision to submit the work for publication.

## Conflict of interest

The authors declare that the research was conducted in the absence of any commercial or financial relationships that could be construed as a potential conflict of interest.

## Publisher’s note

All claims expressed in this article are solely those of the authors and do not necessarily represent those of their affiliated organizations, or those of the publisher, the editors and the reviewers. Any product that may be evaluated in this article, or claim that may be made by its manufacturer, is not guaranteed or endorsed by the publisher.
